# 

**Published:** 1849-09

**Authors:** 


Dr. Hyde's very acceptable communication is received, and will appear in the October No.

